# Controlled Surface Modification to Revive Shallow NV^–^ Centers

**DOI:** 10.1021/acs.nanolett.2c04733

**Published:** 2023-03-16

**Authors:** Jeffrey Neethi Neethirajan, Toni Hache, Domenico Paone, Dinesh Pinto, Andrej Denisenko, Rainer Stöhr, Péter Udvarhelyi, Anton Pershin, Adam Gali, Joerg Wrachtrup, Klaus Kern, Aparajita Singha

**Affiliations:** † Max Planck Institute for Solid State Research, 70569 Stuttgart, Germany; ‡ 3rd Institute of Physics and Research Center SCoPE, University of Stuttgart, 70049 Stuttgart, Germany; § Institute de Physique, École Polytechnique Fédérale de Lausanne, CH-1015 Lausanne, Switzerland; ∥ Wigner Research Centre for Physics, Institute for Solid State Physics and Optics, Budapest, POB 49, H-1525, Hungary; ⊥ Department of Atomic Physics, Institute of Physics, Budapest University of Technology and Economics, Műegyetem rakpart 3, H-1111 Budapest, Hungary; # Center for Integrated Quantum Science and Technology IQST, University of Stuttgart, 70049 Stuttgart, Germany

**Keywords:** NV magnetometry, surface chemistry, quantum sensing, nanopillar, LT-UHV, ODMR

## Abstract

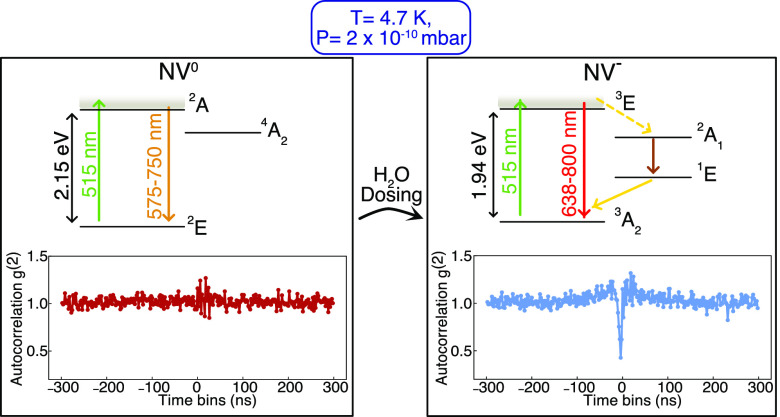

Near-surface negatively charged nitrogen vacancy (NV) centers hold excellent promise for nanoscale magnetic imaging and quantum sensing. However, they often experience charge-state instabilities, leading to strongly reduced fluorescence and NV coherence time, which negatively impact magnetic imaging sensitivity. This occurs even more severely at 4 K and ultrahigh vacuum (UHV, *p* = 2 × 10^–10^ mbar). We demonstrate that *in situ* adsorption of H_2_O on the diamond surface allows the partial recovery of the shallow NV sensors. Combining these with band-bending calculations, we conclude that controlled surface treatments are essential for implementing NV-based quantum sensing protocols under cryogenic UHV conditions.

The nitrogen vacancy (NV) center in diamond is a leading contender for quantum information processing and nanoscale quantum sensing.^1,2^ The application of the NV center as a solid-state quantum sensor spans a wide range, including the investigation of 2D van der Waals magnets with unique spin textures,^3−5^ unraveling superconducting properties at the nanoscale,^6−8^ highly sensitive NMR studies of organic molecules,^9−12^ and recent works of readout and control down to the level of single magnetic molecules.^13^ Notably, all NV-based sensing schemes rely on the unique electronic spin configuration of the negatively charged defect center (NV^–^) and are realized by recording its spin-dependent fluorescence.^14^ However, in near-surface NV centers, it has been shown that several unavoidable interactions often lead to charge transfers, resulting in the formation of the neutral NV^0^ state. This leads to undesired fluorescence quenching and a significant reduction in NV coherence time.^15−20^ NV centers implanted at depths of >10 nm can retain a stable NV^–^ state,^19^ whereas shallow NVs exhibit charge-state instabilities often evidenced as fluorescence blinking.^19,20^ Despite a few attempts to mitigate the issue of charge-state instabilities,^21−28^ a working recipe is still missing for stabilizing near-surface (<10 nm) NV centers without compromising on their performance. In addition, most of these approaches are yet to be explored for single NV centers in a diamond nanopillar, a geometry especially attractive for scanning NV magnetometry.^29^ A controlled, uniform, and robust surface treatment that can successfully promote shallow NV properties in a diamond membrane containing nanopillar arrays should also prove to be effective for hosting shallow NVs in scanning probes, thus improving spatial resolution in scanning NV magnetometry. Furthermore, operations under extreme measurement conditions of ultrahigh vacuum (UHV) in combination with low temperature (4 K) remain elusive, where an abundance of delicate atomic-scale spin systems and physical processes are often investigated.^30−34^

Here, we report the partial recovery of the optical and spin properties in isolated shallow NV centers hosted within individual diamond nanopillars upon controlled surface modifications performed under UHV conditions. Combining these with band-bending calculations, we reveal the correlation between the local changes in the electronic structure of the surface and the charge-state stability of the NV center.

All measurements are obtained from a diamond nanopillar membrane (Figure 1)^35^ under two different conditions: (a) ambient temperature and pressure (NTP) and (b) LT-UHV (*T* = 4 K, *p* = 2 × 10^–10^ mbar). The NV centers are formed at a depth of about (8 ± 3.1) nm by irradiating with 5 keV ^15^N ions (Methods).^36^ The NVs are optically excited by a 515 nm laser. The resulting NV fluorescence is recorded in two ways: (a) after passing through a long-pass (LP) 650 nm filter, the signal is collected by the detection optics (dual APDs mounted in a Hanbury Brown and Twiss geometry) for autocorrelation measurements and (b) after passing through an LP-550 nm filter, the signal is recorded by a spectrometer for emission spectroscopy (further details in Figure 1, Methods, and SI).^37,38^ Notably, given the very high refractive index of diamond, the nanopillar structure on the diamond surface significantly enhances the photon collection efficiency from single NV centers as opposed to shallow NVs within an unstructured diamond membrane.^39^

**Figure 1:**
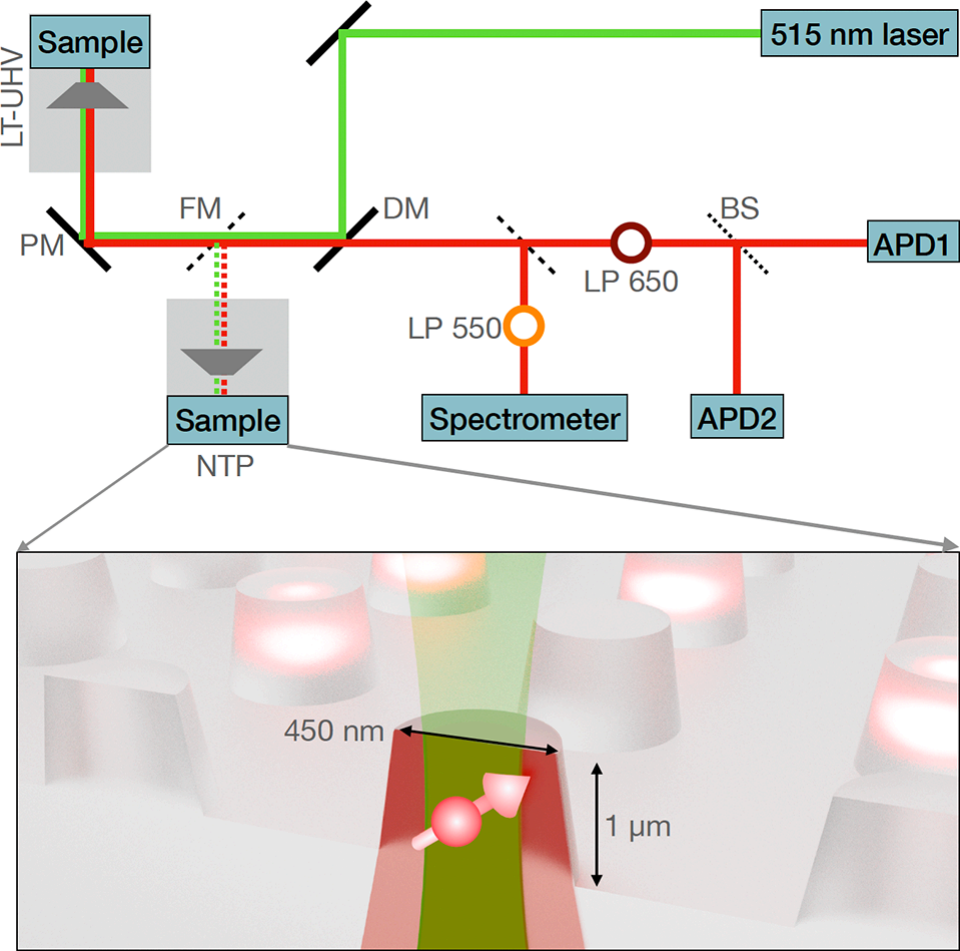
Schematic illustration of the experimental setup. FM, flip mirror; PM, piezo mirror; DM, dichroic mirror; and BS, beamsplitter. Shallow NV centers are hosted in diamond nanopillars. The pillar structure enhances the photon collection efficiency from the single NV centers.

The fluorescence yield of the NV center strongly depends on its charge states, which are characterized by their distinct energy-level distributions.^19^ In shallow NV centers, spontaneous charge-state conversions are often triggered by local imperfections of the diamond surface such as the presence of surface adsorbates and the uncontrolled creation of vacancy clusters and charge traps (created during the NV growth process^40^). The unavoidable interactions and charge transfers with such surface imperfections result in a correlated reduction in the optical and spin properties of the shallow NV centers.^21,41^ In order to verify the environmental impact on the NV center’s charge state, we employed measurements of the emission spectra, autocorrelation, and optically detected magnetic resonance (ODMR) on the same NV centers under NTP and LT-UHV conditions.

An NV^–^ center spectrum is characterized by a zero-phonon line (ZPL) at 637 nm and a phonon sideband with a maximum emission intensity at 690 nm.^42^ In contrast, NV^0^ centers are characterized by a ZPL at 575 nm and have a maximum in the phonon sideband at 640 nm.^42^ Notably, we obtained spectroscopic signatures of the NV^–^ charge state from NTP measurements (black spectrum in Figure 2(a)). Instead, under LT-UHV conditions, the same NV center exhibits a ZPL at 575 nm and a phonon sideband peak at 630 nm, which confirms a strong contribution from an NV^0^ population (red spectrum, Figure 2(a)). This significant modification of the emission spectra reveals a charge-state conversion of the NV center under LT-UHV conditions.

**Figure 2:**
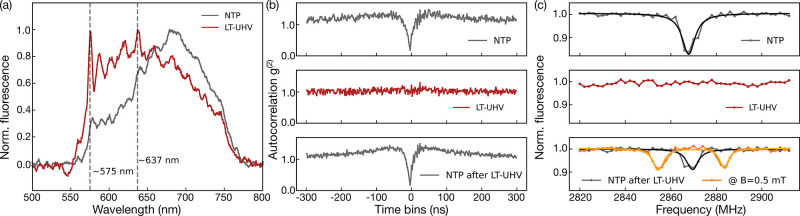
Compared to the measurements under ambient conditions, LT-UHV induces a dramatic degradation of the optical and spin properties of the shallow NV centers, as demonstrated by (a) emission spectra, (b) autocorrelation, and (c) ODMR measurements (dots, measurements; solid lines, Lorentzian fits). All measurements are performed on the same NV implanted at 8 ± 3 nm from the surface (hereafter called NV #1). π pulse lengths used for the pulsed ODMR measurements in (c) are as follows: 250 ns (upper panel), 275 ns (middle panel), 250 ns (lower panel, without field), and 290 ns (lower panel, with field).

The absence of the NV^–^ center under LT-UHV conditions is further supported by our autocorrelation measurements, as depicted in Figure 2(b) for a representative single NV center. At NTP (black curve), we observe a clear antibunching dip at a zero time delay with *g*^(2)^(0) = 0.2. Note that the slight increase in the autocorrelation signal just before the dip indicates a significant NV^–^ population, which can be modeled as a three-level system.^43^ In stark contrast, the antibunching feature completely vanishes under LT-UHV conditions (red curve), which indicates that the majority of the NV^–^ contribution is diminished due to charge-state instabilities, as also realized from emission spectroscopy. We note that the resulting NV^0^ state formed under the LT-UHV condition is also a single photon source. However, the LP650 placed in front of the detection optics (SI section 1) strongly suppresses any signature from NV^0^ in our autocorrelation measurements. This allows us to associate any autocorrelation dip with the presense of a stable NV^–^ center. Note that the NV^–^ center is fully recovered only after it is brought back to the NTP measurement stage.

The distinct electronic and spin properties of the NV^–^ and NV^0^ centers further provide direct evidence of the NV charge state under the two measurement conditions, in terms of electron spin resonance measurements (ODMR spectroscopy). The NV^–^ center forms a spin triplet with a zero-field splitting of 2.87 GHz in the ground state, which is measured as a sharp ODMR line.^14^ By applying an external magnetic field, this resonance is split due to the Zeeman dependence of the sublevels.

In contrast, the NV^0^ center forms a doublet ground state without any spin-dependent fluorescence response. As shown in Figure 2(c), the measurements under ambient conditions exhibit the expected resonance at 2.87 GHz with 18% contrast (black spectrum).^14^ Notably, this resonance completely vanishes under the LT-UHV condition (red spectrum), which recovers only in a subsequent measurement performed under ambient conditions (gray and orange spectra exhibiting a 9% ODMR contrast). For comparability of the measurements, we ensured the use of the same laser and microwave power. The ODMR measured at NTP with a 1.4 mT external magnetic field further exhibits the expected Zeeman splitting of the *m*_*s*_ = ±1 levels as a direct fingerprint of the NV^–^ state. We attribute the slight decrease in the ODMR contrast in the second measurement run under ambient condition to the possible change in the microwave (MW) wire positioning during transfers of the sample to and from the LT-UHV stage. In addition, we note that some NVs have been observed to survive the extreme measurement conditions of LT-UHV, possibly due to their larger implantation depths (additional measurements in Figure S6).

The combined experimental evidence obtained from emission spectroscopy, autocorrelation, and ODMR measurements performed on the same NV centers under the two measurement conditions reveals a dramatic suppression of the NV^–^ population at LT-UHV for shallow implanted NVs. We attribute this to possible adsorption and desorption processes that occur under these extreme measurement conditions which lead to significant changes in the diamond surface, thus strongly affecting the near-surface NVs. Typical candidates which easily adsorb on surfaces under ambient conditions are water molecules. With the relatively large dipole moment of the water molecule (≈ 1.85 D),^44^ a surface-adsorbed water layer at NTP is capable of inducing a strong static electric field which influences the charge-state distribution of the NV defects.^22^

We further link the loss of ODMR contrast at LT-UHV to a change in band bending at the interface, which we describe through a combination of ab initio density functional theory (DFT) calculations and a continuum electrostatic model. To this end, we consider the charge-state dynamics of the NV centers in a bath of nearby electron traps and hole-emitter defects, e.g., vacancy clusters. Our model incorporates the divacancy defects (V_2_) as representative hole emitters together with the substitutional nitrogen (N_s_) donors and NV defects, created during the ion implantation and subsequent annealing processes. The laser illumination generates mobile holes in the valence band through the V_2_^(0)^ → V_2_^(−)^ + h^(+)^ photoionization process. Owing to its large hole capture cross section of 3 × 10^–3^ μm^2^,^45^ the NV center preferentially captures a mobile hole, created during the ionization–recombination dynamics inside the bath of hole emitters. This process causes a charge-state instability and demolishes the optical initialization of the NV^–^ charge state. To be efficient, it requires a thermodynamically stable V_2_^(0)^ charge state, which is in turn affected by the band bending near the surface. Thus, the strength of band bending defines the depth of the region from the diamond surface where the NV^–^ emitter is unstable. We model this band bending using realistic densities of defects and accumulated surface charges and compare the charge transition level (CTL) energy of V_2_^(0)^ with the Fermi level as a function of the depth for a nitrogen-doped sample (Figure 3).

**Figure 3:**
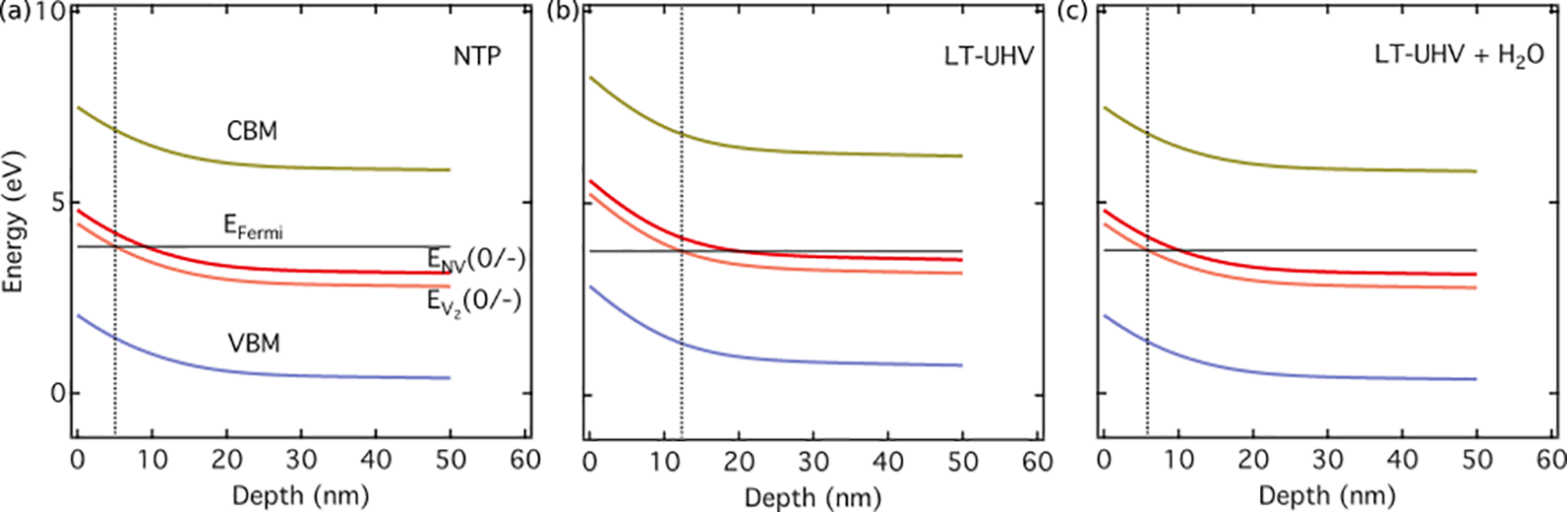
Simulated band bending as a function of depth from the diamond surface. (a) NTP condition with an adsorbed water layer on the surface. (b) LT-UHV condition without the effect of water. (c) Water dosing partially recovering the adsorbed surface layer. Dashed lines show the depth of instability for the NV emitter.

The strength of band bending is deduced from the DFT calculations (details in Figure S7 and the Supporting Information). Our modeling reveals a large positive shift in the potential energy at the interface associated with a dipole moment of the oxygenated surface. The water molecules, adsorbed at the diamond surface, partially compensate for the surface dipole through electron transfer. This translates into overall moderately positive band bending, providing the region of instability for the shallow NV centers (i.e., a region where the CTL of V_2_^(0)^ is above the calculated Fermi level) of 5 nm at room temperature. Under the LT-UHV conditions, the adsorbed water is released from the surface, and the instability region extends to around 12 nm. Controlled water dosing could therefore restore the strength of band bending under ambient conditions, but keep in mind a new charge distribution at low temperature, which results in a slower decay of the energy levels toward the bulk (Figure 3). However, our calculations predict only a small increase in the critical depth to 6 nm at 4.7 K. Hence, a revival of the ODMR contrast due to water dosing should occur for all but the shallowest NV centers (Table S1 and measurement statistics available in the Supporting Information).

In order to verify this and to mitigate the charge-state instabilities under LT-UHV conditions in a controlled manner, we dosed purified water in UHV following several freezing–pumping–thawing cycles (Methods). Subsequently we investigated the effects on the emission spectrum, autocorrelation, and ODMR by measuring on the same precharacterized NV as shown in Figure 4(a)–(c). While the NV fluorescence in the absence of a water layer exhibits a strong NV^0^ ZPL at 575 nm, the latter is significantly reduced upon H_2_O dosing. From the direct comparison of the shift in the phonon side bands, it becomes evident that the deposited water layer helps to promote a stronger relative NV^–^ population (Figure 4(a)). An equivalent change is also observed from the autocorrelation measurements, as illustrated in Figure 4(b). Before water dosing, the NV center did not show any antibunching feature. However, upon water dosing, a clear antibunching dip is observed.

**Figure 4:**
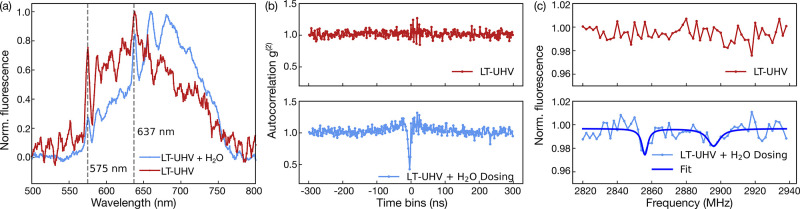
Revival of a shallow negatively charged NV center after water dosing under LT-UHV conditions, as evidenced by (a) characteristic changes in the emission spectrum, (b) the reappearance of the antibunching dip in the autocorrelation signal, and (c) partial recovery of the ODMR signal. All measurements are performed on the same NV center implanted 8 ± 3 nm from the surface (NV #2). π-pulse lengths used for the pulsed-ODMR measurements in (c) are as follows: 120 ns (upper panel) and 150 ns (lower panel).

The partial recovery of the negative charge state of the NV center is also evident from the measured ODMR signals in Figure 4(c). For an undosed sample, no resonance lines were obtained. However, after water is dosed onto the diamond surface, a weak albeit noticeable ODMR signal with a contrast of ∼2% is recorded in a stray magnetic field of 0.7 mT (additional control measurements in Figures S3 and S4).

Notably, a full recovery of the NV^–^ charge state in the LT-UHV environment is not yet achieved via water dosing as evidenced by the relatively low ODMR contrast as well as from the small residual component appearing at 575 nm in Figure 4a (blue spectrum). We identify the latter as a time-averaged measurement of any remaining charge-state dynamics which may be occurring on a time scale much faster than the acquisition time of our emission spectra. Such residual charge-state dynamics as well as the overall partial recovery of the optical and spin properties of the shallow NVs can be attributed to the possible nanoscale local imperfections and/or inhomogeneous distribution of the physisorbed water layer. More robust and controlled surface treatment is expected to overcome these and further enhance the properties of the shallow NVs. Nevertheless, the simultaneous observation of an upshifted phonon sideband, the revival of an autocorrelation feature, and ODMR contrast upon water dosing clearly suggests a relative change in the charge-state population of the NV defect center induced by the adsorption of polar compounds on the diamond surface, thus implying controlled surface modification as a promising viable route for stabilizing shallow NVs, especially for operations under LT-UHV conditions. Note that although similar effects may also be expected for NVs contained within an unstructured diamond surface, the detection efficiency is significantly reduced in such cases simply due to the very high refractive index of diamond.^39^

In conclusion, by combining autocorrelation measurements, ODMR, emission spectroscopy, and theoretical modeling, we confirm that NV centers undergo uncontrolled charge-state modifications under extreme measurement conditions of UHV (2 × 10^–10^ mbar) and low temperature (4.7 K). To the best of our knowledge, this is the first demonstration of instabilities of individual shallow NV centers within diamond nanopillars under these extreme measurement conditions. In addition, we also report that the NV centers exhibit a relative increase in the negative charge state upon *in situ* deposition of water. The latter is expected to indirectly assist the NV charge-state stabilization by hindering the hole emission from the nearby deep acceptor defects. Although the recovery of the negative charge state of the NVs is by far not comparable with that measured at NTP, our experiments clearly indicate that controlled surface treatments play a crucial role in using near-surface NV centers under LT-UHV conditions. Similar or better performance toward charge-state stabilization might be achieved by the controlled surface adsorption of inorganic layers (for instance, via atomic layer deposition processes) or by preparing self-assemblies of robust molecular adsorbates. This altogether brings sensing with shallow NV centers an important step forward, especially for investigating magnetic phenomena at nanometer length scales for samples which are prone to thermal instabilities or to chemical degradation under ambient condition, such as single-molecular systems, thin film superconductors, and skyrmionic structures.

## Methods

The NV layers were created by 2.5, 5, and 10 keV nitrogen ion implantation (^15^N) in an electronic grade (e6) CVD-grown diamond, where the beam energies define the resulting NV center depth^46^ (as shown in Figure S1). The depths were simulated using “SRIM: The Stopping and Range of Ions in Matter” software.^47^ Subsequently the diamond sample was annealed at 950 °C for 2 h. The nanopillar structures were etched in the diamond to enhance the photon collection efficiency.^39^ All measurements reported in this work are performed with NVs implanted using a 5 keV beam energy.

In order to perform measurements on a clean surface, the diamond membrane was cleaned and oxygen terminated by triacid boiling (HNO_3_/HSO_4_/HClO_4_) at 200 °C for 6 h. Afterward, the diamond sample was glued onto a sample holder with an LT-UHV compatible varnish. After leaving it in the preparation chamber maintained at 2 × 10^–10^ mbar for 12 h, the sample was finally transferred into the cryogenic UHV measurement head (<2 × 10^–10^ mbar).

The water dosing was performed in the preparation chamber of the LT-UHV setup. Distilled H_2_O was utilized after purification by up to 10 rounds of a freeze–pump–thaw cycle.^48,49^ The freeze–pump–thaw cycle is performed as a cleaning procedure to eliminate paramagnetic impurities dissolved in water (such as oxygen). This is a crucial step as impurities present in water may otherwise result in a broad resonance signal and may introduce other spurious effects (as shown in SI Figure S7). In the absence of a direct quantitative analysis of the surface quality, for example, via a high-resolution imaging technique, such a cleaning step is crucial to ensure reproducible surface treatments and the repeatability of our measurements. A stainless steel needle attached to the high-precision leak valve which is connected to the water reservoir was placed in close proximity to the diamond surface (within submillimeter distances). The H_2_O was introduced through the high-precision leak valve into the preparation chamber at 5 × 10^–7^ mbar for 120 s. The dosing was performed directly after transferring the sample from the 4 K measurement head to the preparation chamber, leading to the reasonable assumption of a cold diamond surface during dosing and therefore strong adsorption of the water molecules. Subsequently, the sample was transferred to the LT-UHV environment for further measurements.

Based on the partial pressure of our preparation chamber during the water dosing process and the dosing time, we estimate an upper bound of ∼5 monolayers (MLs) of adsorbed water, which is equivalent to a 1.25-nm-thick water layer on the diamond surface.^50^ Here 1 ML is defined as the entire diamond surface being uniformly coated with water molecules. Note that this estimate does not take into account the sticking coefficient of the diamond surface, which may further reduce the coverage even up to a factor of 2.^51^ Furthermore, the estimate is based on the assumption that every water molecule leaving the nozzle of the precision leak valve is adsorbed onto the cold diamond surface. Experimentally, we have verified that the presented data corresponds to the maximum possible water coverage on the diamond surface, as dosing times longer than 120 s do not improve the NV properties any further (Figure S8). This also suggest that interlayer hydrogen bonding may not have a discernible role in determining the shallow NV spin properties for water layer thicknesses ≤1.25 nm.

Note that the central motivation behind performing *in situ* surface treatment under clean UHV conditions (*p* < 2 × 10^–10^ mbar) is to be able to gain precise control over the diamond surface quality. Dosing under ambient conditions does not allow the same repeatability and control as in that case several different adsorbate molecules from the ambient air would already stick to the diamond surface without any hindrance. In addition, the entire dosing process has to be performed within a relatively short time so that the probability of any unwanted residual gas molecule sticking to the diamond surface from within our UHV preparation chamber can be kept to a minimum. For our measurement sequences, this is possible only when the dosing is performed swiftly on a cold substrate.

Ab initio calculations were performed by using the PBE functional^52^ in conjunction with the D2 dispersion correction scheme, implemented in the VASP package.^53^ A projector augmented wave method with a kinetic energy cutoff of 370 eV was used. In our simulations, we used a slab of (100) diamond terminated with the hydrogen, hydroxyl, and ether surface groups.^41^ The diamond slab was put in contact with 74 water molecules and 1.9 nm of vacuum in a simulation supercell of 1.0097 × 1.0097 × 5.3 nm^3^. A representative configuration was captured from the ab initio molecular dynamics trajectory of 10 ps and was subsequently relaxed to a local minimum.

We calculate the depth dependence of the electric potential *V*(*x*) inside a diamond by numerically solving the boundary value problem of Poisson’s equation
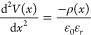
1where ρ(*x*) is the total charge density consisting of free space charges and defect acceptors and donors, ε_0_ is the vacuum permittivity, and ε_*r*_ = 5.5 is the relative permittivity of diamond. There are two boundary conditions to satisfy: first, the electric field vanishes in the bulk, and second, the potential at the diamond surface is set according to the DFT calculation results. The accumulated surface charge density is calculated from the charge neutrality condition from the total charges inside the diamond. The band-bending curves in Figure 3 are simulated for accumulated surface charge densities of −0.041, – 0.051, and −0.042 *e*/nm^2^ for panels a–c, respectively. We use the following characteristic energies in the bulk during the simulations, given relative to the valence band maximum. *E*_C_ = 5.45 eV, *E*_NV_(0/−) = 2.75 eV, *E*_NV(+/0)_ = 0.9 eV, *E*_Ns(+/0)_ = 3.75 eV, , and  are the energy levels for the conduction band minimum, NV center acceptor and donor levels, substitutional nitrogen donor level, and divacancy donor and acceptor levels, respectively. We assume Gaussian defect density profiles for the implantation, centered at a 10 nm depth with a variance of 10 nm. The peak implanted defect concentrations are ρ_Ns_ = 15 ppm, ρ_NV_ = 3 ppm, and . The acceptor and donor densities are calculated from the Fermi–Dirac statistics with respect to the Fermi level in the bulk.

## Supporting Information Available

Details of the experimental setup, additional control measurements, and the details regarding DFT calculations (PDF)
