# Supplementary material for: Controlled Surface Modification to Revive Shallow NV– Centers
Source: Nano Lett. 2023 Mar 16;23(7):2563–9. doi: 10.1021/acs.nanolett.2c04733 (PMC10103335; doi:10.1021/acs.nanolett.2c04733)
Supplement: Supplementary file 1 — nl2c04733_si_001.pdf [file nl2c04733_si_001.pdf]

Supporting information: Controlled surface modification to revive shallow NV⁻ centers

Jeffrey Neethi Neethirajan,[†] Toni Hache,[†] Domenico Paone,[†,‡] Dinesh Pinto,[†,¶] Andrej Denisenko,[‡] Rainer Stöhr,[‡] Péter Udvarhelyi,[§,∥] Anton Pershin,[§,∥] Adam Gali,[§,∥] Joerg Wrachtrup,[‡,†] Klaus Kern,[†,⊥] and Aparajita Singha[*,†,#]

†*Max Planck Institute for Solid State Research, 70569 Stuttgart, Germany*

‡*3rd Institute of Physics and Research Center SCoPE, University of Stuttgart, 70049 Stuttgart, Germany*

¶*Institute de Physique, École Polytechnique Fédérale de Lausanne, CH-1015, Switzerland*

§*Wigner Research Centre for Physics, Institute for Solid State Physics and Optics, Budapest, POB 49, H-1525, Hungary*

∥*Department of Atomic Physics, Institute of Physics, Budapest University of Technology and Economics, Műegyetem rakpart 3., H-1111, Budapest, Hungary*

⊥*Institute de Physique, École Polytechnique Fédérale de Lausanne CH-1015, Switzerland*

#*Center for Integrated Quantum Science and Technology IQST, University of Stuttgart, 70049 Stuttgart, Germany*

E-mail: mailto:a.singha@fkf.mpg.de

Experimental Setup and NV depth profile

The experimental setup is illustrated in Fig. 1 of the main manuscript. The 515 nm laser (Toptica iBeam-Smart-515) is guided (a) to an ambient piezo controlled stage (Piezosystem

Jena T-404-01D) or (b) to the LT-UHV setup.[1] For both cases the resulting NV center's fluorescence passes through a dichroic mirror and is recorded with two crossed avalanche photodiodes (APDs) (Excelitas SPCM-AQRH-14) enabling photon autocorrelation measurements. In front of these photon detectors, a pinhole (spatial filter) with a diameter of 75 μm is mounted. Additionally, a 650 nm longpass (LP) filter is implemented for enhanced selectivity of the emission from the negatively charged centers.

In both measurement conditions, NV center spin manipulation is realized with an integrated microwave source (Vaunix Lab Brick LSG-402). ODMR spectroscopy is performed in a pulsed measurement scheme to maximize the NV center spin contrast. Confocal scans are obtained by moving the sample holder (piezo mirror) in ambient (LT-UHV) conditions over the fixed laser spot (fixed sample) (see confocal scan in ambient condition in Fig. 2). A second flip mirror is implemented between the pinhole and photon counting devices for guiding the NV center emission to a high performance spectrometer (QEPro-FL). In this particular collection path, an LP 550 nm filter is mounted in front of the spectrometer, for recording NV^0 and NV^- contributions simultaneously.

The depths of the implanted NVs were simulated using "SRIM: The Stopping and Range of Ions in Matter" software[2] (as shown in Fig. 1). High implantation energies of 10 keV lead to relatively deep NV centers ($\approx$ 15 nm) within the diamond matrix while lower energies of 5 keV (2.5 keV) result into shallow NV defects at $\approx$ 8 nm ($\approx$ 4.6 nm).[3]

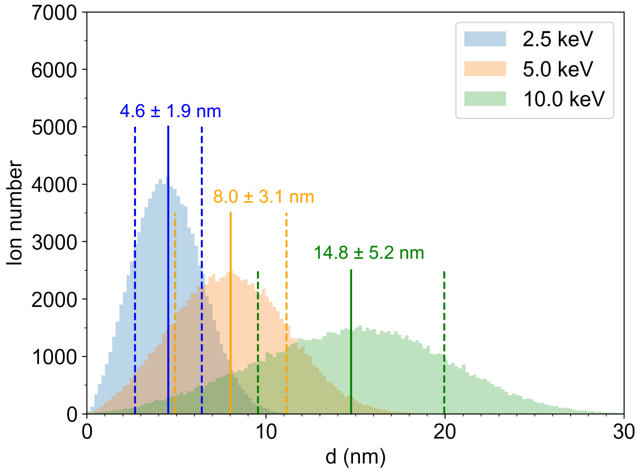

Figure 1: Simulation of the depth distribution of ^{15}N ions for the given implantation energies. For each energy 99999 ions were simulated. The density of the diamond was assumed to be 3.51 g/cm^3. The vertical solid lines indicate the mean depth. The dashed lines indicate the straggling (square root of the variance).

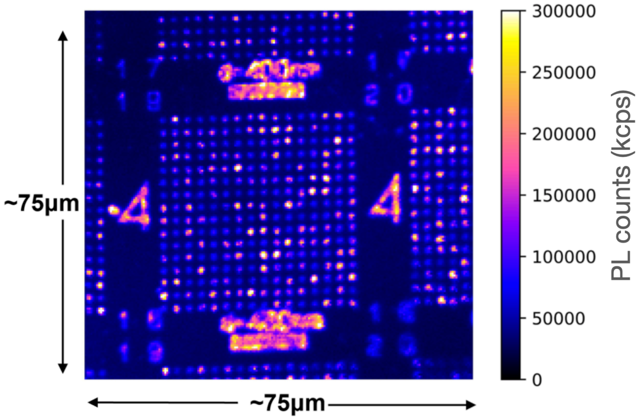

Figure 2: The confocal scan shows an area of our single-NV implanted diamond membrane measured at NTP. The diamond sample is divided in different fields hosting individual nanopillars. The bright spots in a given nanopillar indicate the presence of single NV-centers. Lithographically written markers allow for easy identification of a specific nanopillar with NV center for comparing the properties at NTP and UHV-cryogenic conditions.

LT-UHV setup consists of three distinct chambers. For transferring the sample from ambient conditions, it is first placed into the loadlock chamber which is maintained at a high vacuum of $\approx 2{\cdot}10^{-7}$ mbar. Afterwards the sample is transferred into the preparation chamber

maintained at $\approx 2 \cdot 10^{-10}$ mbar. Finally, the sample is transferred into the main chamber which contains the measurement head at cryogenic 4.7 K, $2 \cdot 10^{-10}$ mbar UHV conditions and in which the NV center characterization measurements are performed. All transfers are performed without cracking the vacuum. In addition, this chamber configuration allows for sample transfers without heating up the He bath cryostat.

Effects of controlled surface-modification and additional control measurements

Similar to the case of NV #2 reported in Fig.3 of the main manuscript, the beneficial effect of water dosing has also been observed in three additional NV-centers (named as NV #1, NV #3, NV #4). Fig. 3 and Fig. 4 illustrate this from the evolution of the autocorrelation and ODMR measurements respectively, before and after water dosing.

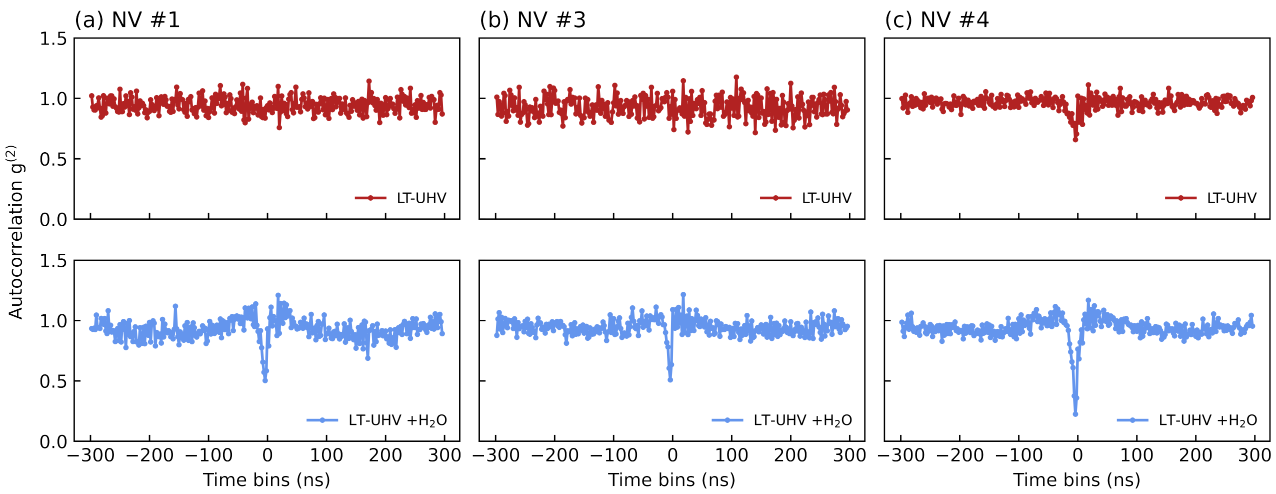

Figure 3: Revival of three additional NVs upon water dosing as evidenced by autocorrelation measurements performed at LT-UHV condition. Note that NV #1 is the same as reported in Fig. 2 of the main manuscript.

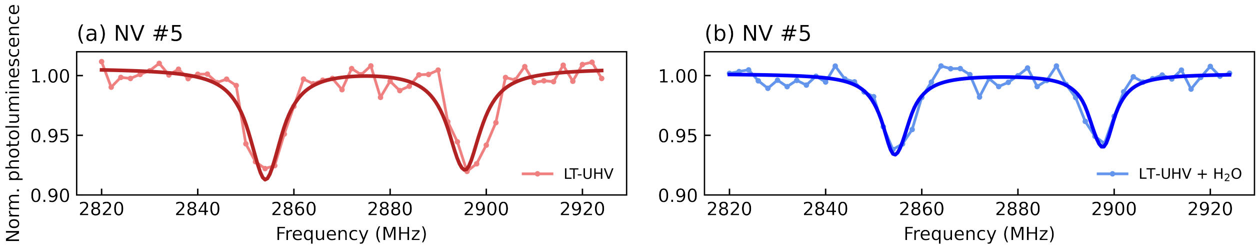

Figure 4: ODMR measurements on NV #1 at 0.7 mT and NV #4 at 0.6 mT stray magnetic field acquired before and after water dosing. Pulse lengths used are 250 ns (upper panel) and 500 ns (lower panel) in (a) and 250 ns (upper panel) and 120 ns (lower panel) in (b) respectively. Corresponding ODMR measurements on NV #3 was not successful.

In order to verify the effects of water, as well as to perform additional control experiments, we have performed surface treatments by dosing nitrogen gas. Nitrogen is a non-polar molecule which does not posses any dipole moment and thereby it is not expected to help shallow NVs revive. Our measurements illustrated in Fig. 5 are in good agreement with this as evidenced from the autocorrelation and ODMR measurements on the same NV. Note that similar to the case of water dosing, nitrogen gas was also dosed for 120 s at 5×10^{-7} mbar partial pressure in the preparation chamber using another high-precision leak valve which was only dedicated for the nitrogen gas line. In addition, dosing was performed on to a cold sample by transferring it from the cold measurement head to the preparation chamber without cracking the vacuum.

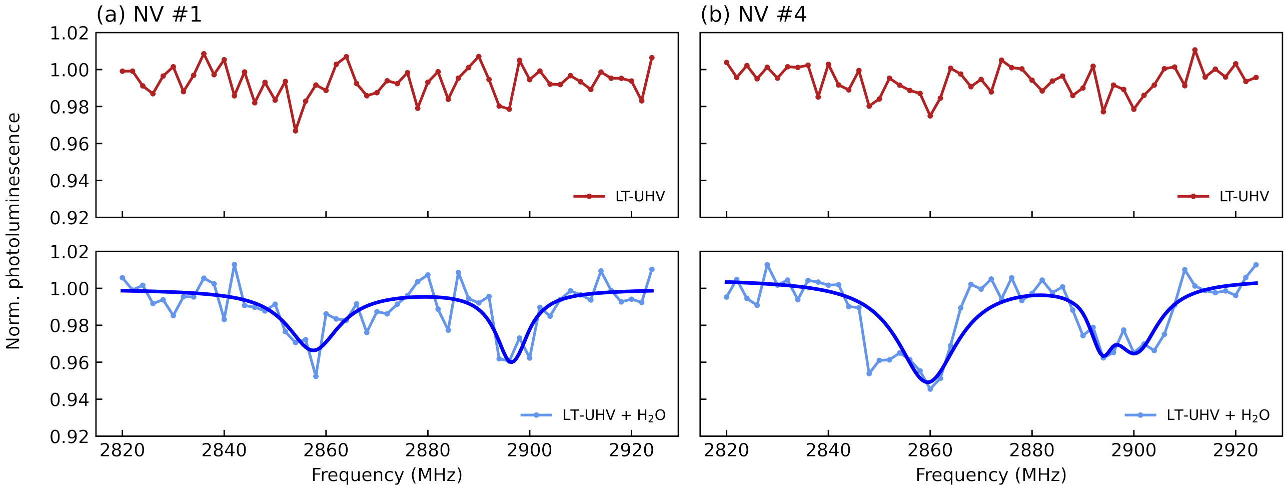

Figure 5: Effects of nitrogen vs water dosing on the same NV-center as evidenced by (a) autocorrelation, (b) ODMR measurements (in stray magnetic field of 0.7 mT). Note that this is the same NV as reported in Fig.3 of the main manuscript.

As illustrated in Fig 1, the NVs implanted with 5 keV nitrogen beam will possess a mean depth of 8 ± 3 nm. Given significantly large width of the depth distribution, we have also encountered single NVs within the same implantation field which are insensitive towards modifications made to the surface. We attribute such observations to a possibly longer distance of those NVs from the surface. Fig. 6 exhibits one such case of a single NV-center which survived the LT-UHV condition. Consequently it also does not exhibit any effect upon water dosing.

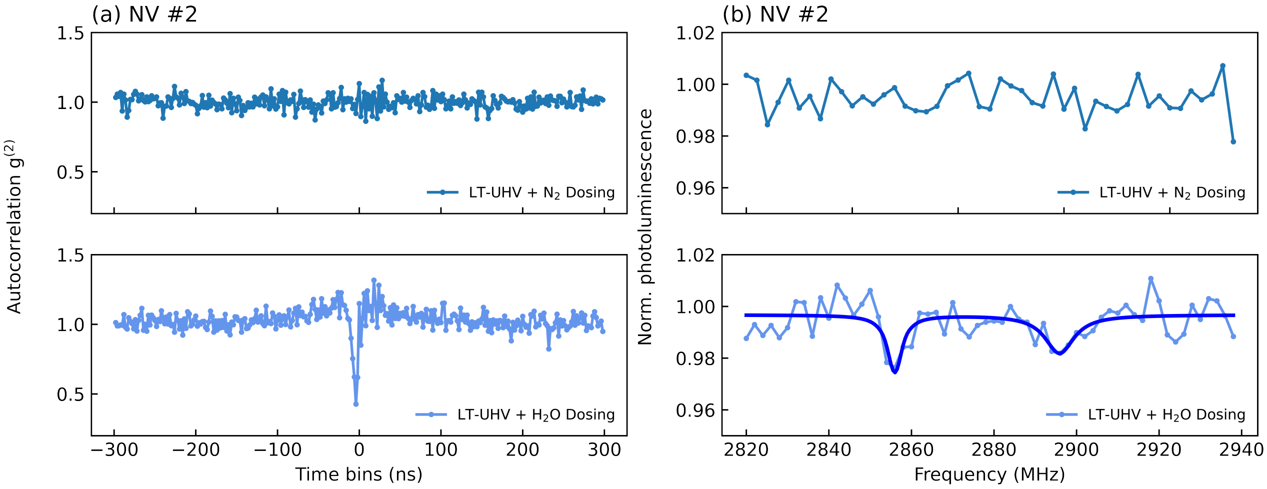

Figure 6: An example of a single NV center that survives the LT-UHV measurement condition and thereby does not show any improvement upon further water dosing as evidenced by ODMR measurements in a stray magnetic field of 0.74 mT in (a) and 0.77 mT in (b). Pi-pulse length used for the pulsed-ODMR measurements is 111 ns.

Note that we needed to perform at least ten rounds of freezing-pumping-thaw cycles for purifying the water prior to dosing, in order to ensure repeatable surface treatments and reproducibility of our measurements. This is very crucial as impurities present in water may otherwise result in a broad resonance signal and may introduce other spurious effects such as reduction in ODMR contrast. Two such cases are illustrated in Fig. 7 where only four cleaning cycles were performed prior to water dosing.

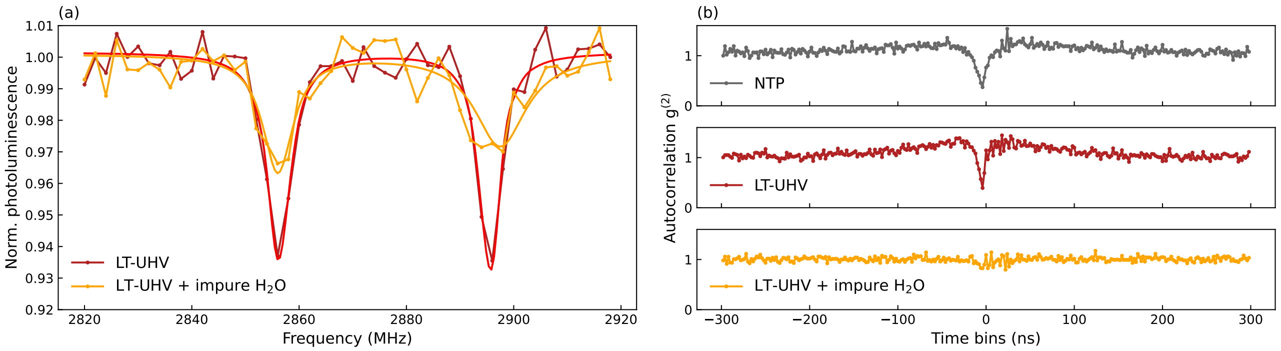

Figure 7: Example of two single NV centers one of which exhibits reduced ODMR contrast in (a) while the other one shows reduced autocorrelation dip at zero time delay in (b), upon dosing water without a thorough cleaning cycle. The autocorrelation measurements are obtained from an NV in the 5 keV implanted region whereas the ODMR measurements are performed on an NV from 10 keV implanted region. The Pi-pulse lengths used for the ODMR measurements are 140 ns and 100 ns respectively.

We estimate an upper bound for the water coverage as 5 ML (equivalent to $\leqslant$ 1.25 nm thickness) for all data presented in this work. Moreover, we have verified that the resulting effects on the shallow NV-centers do not change in any discernible manner even after dosing for a longer time (see Fig. 8). Therefore, assuming an ideal sticking efficiency of water on to the cold diamond substrate, these observation indicate that interlayer hydrogen bonding does not play any distinctive role in determining the shallow NV properties for water thickness $\leqslant$ 1.25 nm.

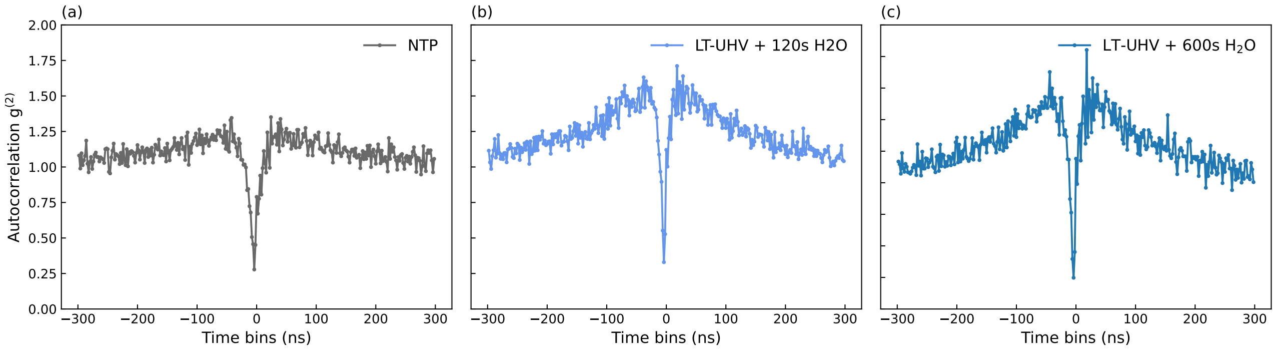

Figure 8: An example of sequential water dosing onto a single NV center, showing no further discernible improvement in the autocorrelation dip at zero time delay beyond 120 seconds of dosing. All measurements are performed on NV#4.

Finally, in order to understand and disentangle the effects of low temperature in determining the charge state instabilities of shallow NV centers, we have performed additional measurements at room temperature while keeping the diamond sample inside the LT-UHV measurement head maintained at $\mathsf{P} = 1\times10^{-9}$ mbar. Based on our measurements of autocorrelation and emission spectroscopy on the same 2.5 keV implanted NV-center, we conclude that radical changes occur in the NV-properties when the sample is brought from NTP conditions to UHV condition at room temperature (Fig. 9). These measurements are also in agreement with our theoretical model, which does not include any explicit temperature dependence. Based on these observation, we conclude that the cryogenic temperature itself does not play any direct role in the water desorption process and subsequent instabilities in shallow NVs. However, addition of water in low temperature redefines the surface charge distribution, which eventually influences the stability zone for the shallow NVs, as shown in Fig. 3 of the main manuscript.

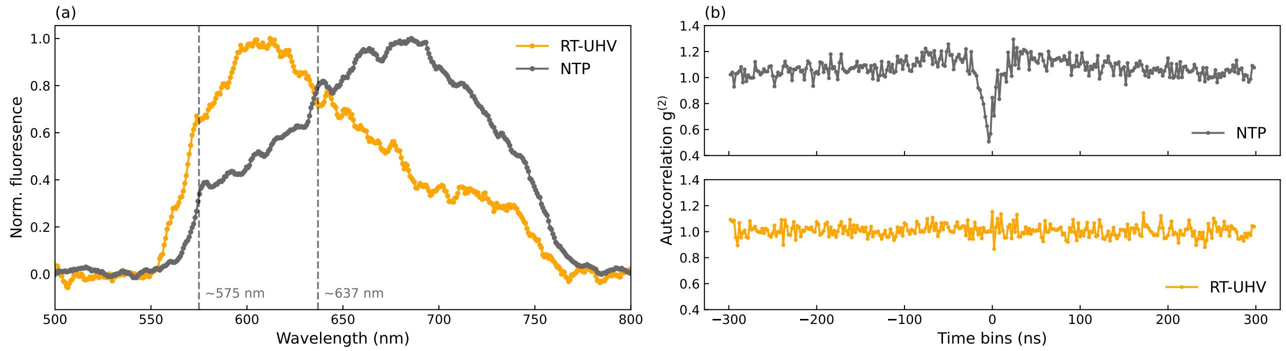

Figure 9: UHV measurements performed at room temperature and $\mathsf{P} = 1 \times 10^{-9}$ on a single NV exhibit a clear downward shift in the emission spectra in (a) as well as a drastic change in the autocorrelation dip at zero time delay in (b). All measurements are performed on an NV from 2.5 keV implanted region.

Controlled surface modification and depth dependence of the shallow NV properties

We have measured on 9 NVs from 2.5 keV, 9 NVs from 5 keV, and 7 NVs from 10 keV implanted regions. The table given below provides the statistics of the investigated NVs in different measurement conditions.

	2.5 keV	5 keV	10 keV	Measurement condition
stable/total	9/9	9/9	7/7	NTP
stable/total	1/9	5/9	5/7	LT-UHV
stable/total	1/9	9/9	5/7	LT-UHV + H_2O dosing

The notation "stable" stands for the existence of a strong NV^- charge state population defined by the measured autocorrelation curves, ODMR, and emission spectra (as presented in the main manuscript). The number of NVs that remain stable in LT-UHV stage even without any surface treatment, increases with increasing implantation energy. We attribute this observation to their increasing effective distances (vertical and/or horizontal) from the diamond surface. Furthermore, we note that nearly 50% of the investigated NVs in the 5 keV implanted region becomes unstable under LT-UHV condition prior to water dosing. The revival of those NVs upon controlled water dosing has been presented in the previous sections. The strongest impact, however, is observed for the NV centers implanted with 2.5 keV nitrogen beam. NVs in this region have an average depth of 4.6 nm (Fig.1). Therefore, in very good agreement with our simulated threshold for the band bending cross over region of 6 nm (Fig. 3c of the main manuscript), these NVs remain unstable even upon water dosing in LT-UHV. In contrast to the NVs from the 2.5 keV and 5 keV implantation regions, the ones from the 10 keV field are generally more stable due to their larger effective distance from the diamond surface. The absence of any change in the remaining two NVs in the 10 keV implantation region can be attributed to the statistical nature of the resulting NV depth,

which may also lead to very few near-surface NV centers ($\leqslant$ 6 nm) even for implantation with high energy beams.

Note the horizontal position or the proximity to the periphery of the nanopillar may also play the same role in charge state conversion. However, it is challenging to control the horizontal position of the NV within a nanopillar from fabrication point of view. In addition, it is difficult to identify and rule out such cases from measurements presented within the scope of this work. Nevertheless, also in those cases we expect our approach with water dosing to have similar effects, as long as the NV-center is deeper than 6 nm from any diamond surface.

Density functional theory calculations

We have directly simulated the electrostatic profile of diamond/water interface by means of the density functional theory. To this end, we considered two models of oxygenated diamond, namely, an unsaturated asymmetric slab and a fully saturated symmetric diamond. The oxygenated diamond model involves a carboxyl group with a deep acceptor level in the gap that pins the Fermi-level at the surface. This model represents the surface defects with deep acceptor levels. The asymmetric slab provides an electrostatic potential curve to evaluate an absolute value of vacuum level shift (VLS) due to the surface dipole (determines a boundary condition for solving the Poisson's equation). The symmetric slab was used to correctly model the effect of a water layer, which was exposed to one side of the slab, see Fig.10(a). The electrostatic potential across the asymmetric slab is shown in Fig.10(b). By comparing the potential on both sides, we deduce a VLS of 2.84 eV, pointing to an upwards band bending. Note that the value was obtained with an extended 2 nm slab to properly account for the electronic screening effect. The large interfacial dipole originates from a charge transfer between the carbon atoms and the oxygen-related groups. The Bader charge analysis for the symmetric slab shows that effectively two electrons per carbon atom are

transferred to the acceptor groups; thereby the holes are delocalized over the first two layers of carbon atoms in diamond, see Fig. 10(c). The resulting charge redistribution gives rise to an interfacial dipole of -1.58 **e**Å, which sets the value of the calculated VLS.

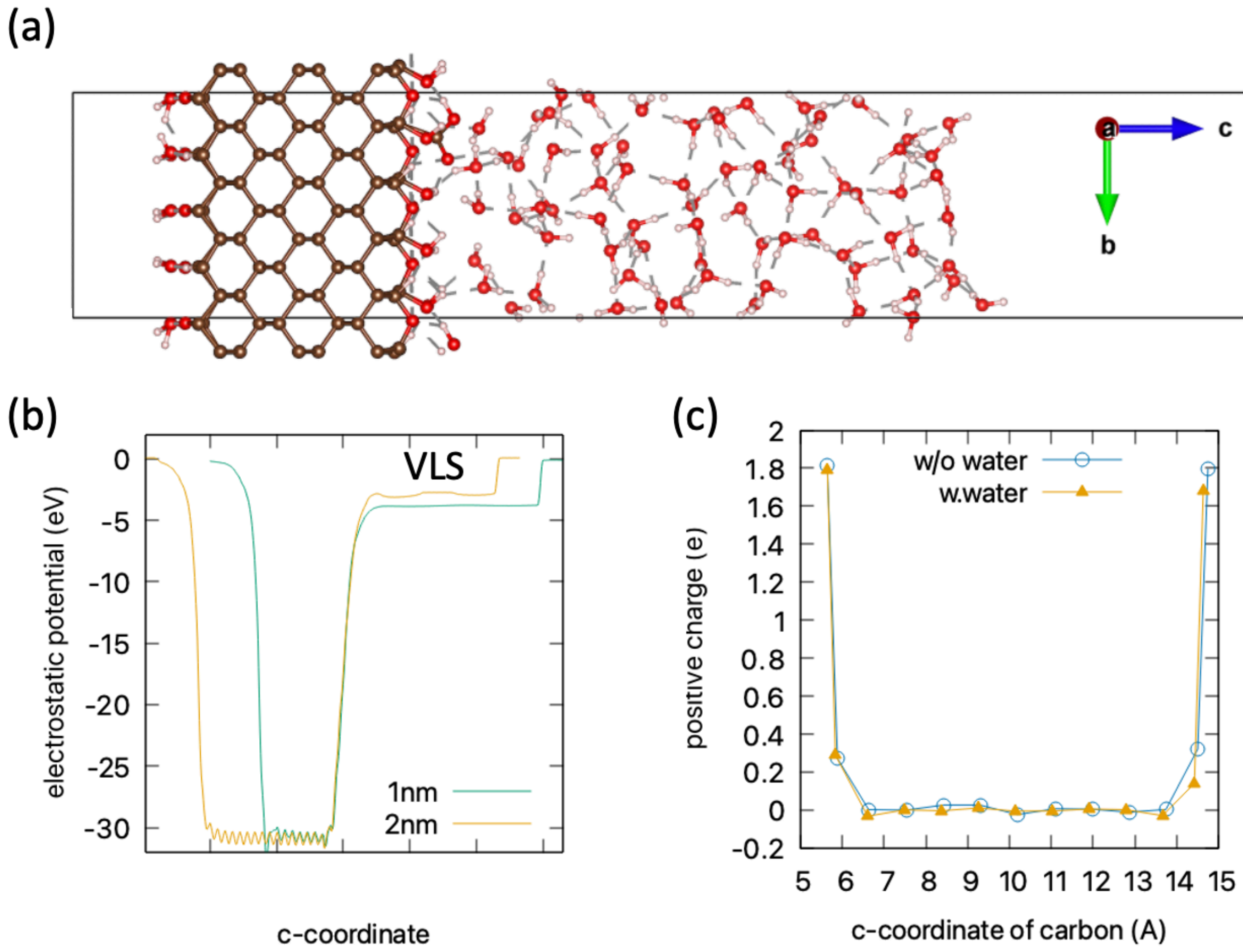

Figure 10: (a) Structure of a symmetric diamond slab together with water and vacuum. Color code: grey is for carbon, red is for oxygen, white is for hydrogen. (b) Electrostatic potential across the asymmetric slabs of 1 nm and 2 nm. The step in the potential corresponds to the VLS. (c) Partial positive charges at carbon atoms, computed in the symmetric slabs as a function of the depth. The blue curve (w/o water) was calculated in vacuum, and the yellow curve (w. water) reflects the effect of the solvent taken in the configuration from (a).

We now estimate how the interfacial dipole changes due to water exposure. Here, we only focus on the bond dipole, i.e., on a contribution from the charge transfer between water and the diamond surface. In fact, the total dipole moment, computed for the slab in Fig. 10(a), also accounts for the dipoles from the diamond surface and water layer. Those

were calculated for the isolated parts of the system and subsequently subtracted from the total value. Note that this assumption implies that the adsorbed water layer is amorphous, i.e., it does not develop a relevant dipole moment. As a result, we found that the interfacial dipole reduces to -1.13 $\mathbf{e}$Å, owing to an electron transfer from water to the acceptor groups. This dipole corresponds to the VLS of 2.05 eV in the presence of water. The effect is also visible from a reduction of the Bader charges in the slab on the side of water, yielding a comparable decrease of the surface dipole. Therefore, we conclude that water develops a bond dipole, which reduces the effect of the surface groups by about 30%.

References

(1) Schaefer-Nolte, E.; Reinhard, F.; Ternes, M.; Wrachtrup, J.; Kern, K. A diamond-based scanning probe spin sensor operating at low temperature in ultra-high vacuum. *Review of Scientific Instruments* **2014**, *85*, 013701.

(2) Ziegler, J. F.; Ziegler, M.; Biersack, J. SRIM – The stopping and range of ions in matter (2010). *Nuclear Instruments and Methods in Physics Research Section B: Beam Interactions with Materials and Atoms* **2010**, *268*, 1818–1823, 19th International Conference on Ion Beam Analysis.

(3) Ishizu, S.; Sasaki, K.; Misonou, D.; Teraji, T.; Itoh, K. M.; Abe, E. Spin coherence and depths of single nitrogen-vacancy centers created by ion implantation into diamond via screening masks. *Journal of Applied Physics* **2020**, *127*, 244502.
